# The haves and the have nots

**DOI:** 10.7554/eLife.01515

**Published:** 2013-11-19

**Authors:** Eve Marder

**Affiliations:** Department of Biology and the Volen National Center for Complex Systems, Brandeis University, Waltham, United Statesmarder@brandeis.edu

**Keywords:** Living science, science policy, careers in science, funding

## Abstract

As the equipment needed to perform state-of-the-art research in many areas of biology becomes ever more expensive, **Eve Marder** worries that researchers in less wealthy institutions might be left behind.

I don’t remember when I first realized that some people have more money than others. My parents were relatively poor when I was young, and I learned to read in a small town in New Jersey that had no high school but hordes of children. When I was 10 we moved to Irvington, a small town outside New York City that did have a high school. Irvington then was a village of contrasts: many of my school mates were children of the town tradesmen, and helped out in their parents’ businesses after school. Nonetheless, much of the land in Irvington belonged to wealthy families who, years previously, had built enormous stone mansions (or so they seemed to those of us who climbed over stone walls to wonder at these edifices), and the taxes paid by these wealthy landowners educated the rest of us.

As I grew older I became aware of the dramatic discrepancies in wealth and educational opportunity in the United States and abroad. But, as a child of the 1960s, nothing prepared me to anticipate what we are witnessing today: increasing wealth discrepancies, between workers and their CEOs, or between the rapid improvements in living standards experienced in some parts of the world, while other regions suffer in ways that are difficult to comprehend given the wealth shared by some of us.

So, what does this have to do with science? It is surprising how many times in the past few years a colleague has used the term ‘the haves and the have nots’ in talking about grant funding for science. Not too long ago, while I was visiting another institution, a talented and productive mid-career scientist used this term to express his fear that he might not be able to renew his grants, even though his science was more exciting than it had ever been. The fear of an uncertain future was doing him more damage than the actual reality of his funding (which was more than adequate to support his work). Of course his fear was not irrational: when adjusted for inflation, funding for research is falling in many countries around the world.

**Figure 1:**
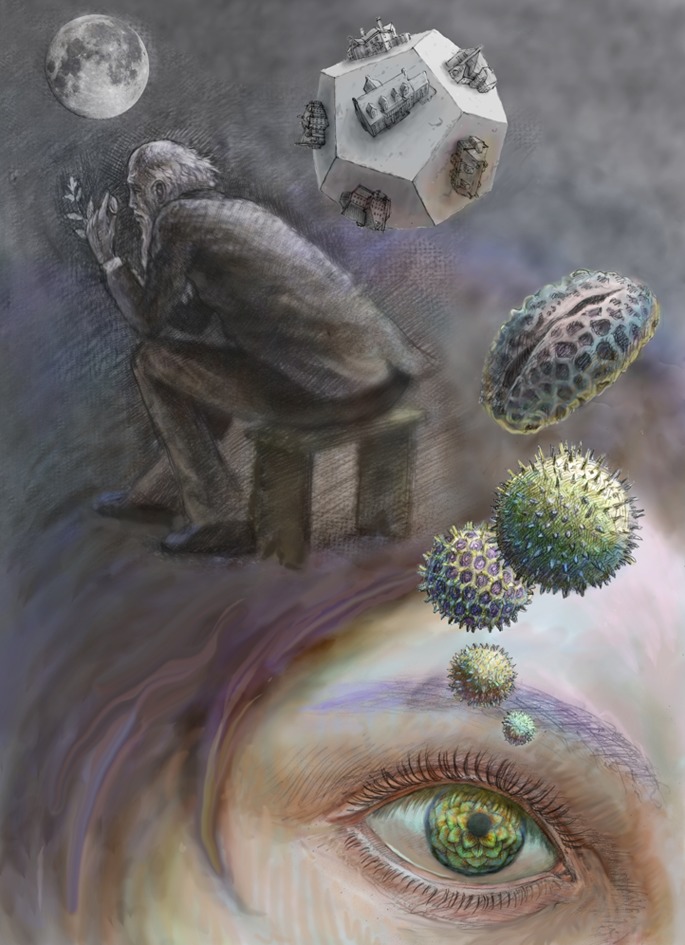
It is important to leave space for thoughtful and creative individuals as access to expensive equipment becomes more and more important in research.

ILLUSTRATION: BEN MARDER

The phrase ‘the haves and the have nots’ is now commonly used by many to describe the resource advantage that some investigators have over their colleagues. It is increasingly the case that state-of-the-art biology requires access to big-ticket items. A laboratory with multiple two-photon microscopes or access to an expensive electron microscope can do experiments that are not possible for a less well-resourced laboratory. And of course, the best resourced laboratories are often clustered in a few rich and prestigious institutions around the world.

The best resourced laboratories are often clustered in a few rich and prestigious institutions.

So, despite all of the rhetoric about resource and reagent sharing, the reality of doing science has become that who you are and where you work play an increasingly larger role in the kind of science you can do. Additionally, funding agencies, donors and foundations disproportionately award grants to individuals and groups at elite institutions, thus amplifying the inequalities. Good ideas and imagination will always trump research dollars or euros or yen, but there are creative experiments that only well-funded laboratories can attempt, and many of our most talented young scientists may be denied access to the resources available to more privileged competitors and colleagues.

Many talented scientists may be denied access to the resources available to more privileged competitors and colleagues.

Study sections and grant panels have always faced the dilemma of how to allocate funds among competing applications. Some argue that putting additional resources into the hands of the ‘best people’ is the optimal strategy. Others argue that the vitality of the scientific enterprise is best served by spreading the wealth around. Like many issues in life, this is a complex calculus: once a laboratory has ‘enough’ money and resources, additional resources may result in diminished productivity per person per unit of funding. And it can lead to diminished happiness among lab personnel who are now competing for their mentor’s attention. On the other hand, blindly spreading resources around may result in no one having adequate money and/or personnel to do important but expensive experiments.

For quite a long time in modern science we have profited from a haphazard mixture of large and small laboratories, operating on a range of budgets. Sometimes the big new ideas came from big labs, and sometimes they came from the brilliance of a small laboratory unburdened by too much money but blessed with clever and thoughtful investigators. The diversity of our laboratories allowed scientists and science of all kinds to flourish, creating a variety of apprenticeships for young scientists, some of whom thrive in small and more focused environments, while others benefit from the opportunities of large science. But we seem to have approached a tipping point: the remarkable new technologies that are now available are often expensive.

While the United States is decreasing its federal support for research in the biological sciences, many other countries around the world are increasing their investments in science. These increased investments have many important benefits: outstanding science is being done, and opportunities for young scientists in their native countries and elsewhere are appearing. Some of the new positions being created are following the best traditions: young scientists given autonomy to set their own research agendas. In other instances, the benefits of the increased investment in science are threatened by administrative structures or traditions that fail to allow sufficient autonomy at the individual investigator level to foster creativity.

Extraordinary individuals do emerge from impoverished environments. In principle, innovative science can emerge from under-resourced laboratories. But, do we wish to return to the eras in history in which science was done by men of wealth and privilege who had the time and the financial resources to indulge their interests? It would be very sad if we lost the many benefits of the democratization and decentralization of science, just at the very moment when we are poised to make exceptional discoveries. Indeed, as the old explorers needed sponsors to outfit their ships for voyages across dangerous and unknown seas, today the new explorers into the unknowns of biology require sponsors who understand the serendipity of scientific exploration, and who realize that sending multiple ships into the unknown is more likely to succeed than sending a small number of lone voyagers.

